# Safety and Efficacy of Tree Pollen Specific Immunotherapy on the Ultrarush Administration Schedule Method Using Purethal Trees

**DOI:** 10.1155/2014/707634

**Published:** 2014-03-24

**Authors:** Andrzej Bozek, Krzysztof Kolodziejczyk, Jerzy Jarzab

**Affiliations:** ^1^Clinical Department of Internal Disease, Dermatology and Allergology, Medical University of Silesia, M Sklodowskiej Curie 10, 41-800 Zabrze, Poland; ^2^Outpatient Allergology Clinic, Lompy 16, 40-038 Katowice, Poland

## Abstract

*Background*. Specific immunotherapy (SIT) with an ultrarush administration schedule with Purethal for tree pollen allergens has been evaluated to assess its efficacy and safety.* Methods*. The study group consisted of 22 patients with symptoms of allergic rhinitis and confirmed allergy to tree pollens. Patients were randomized and given an administration schedule of either ultrarush therapy or conventional preseasonal SIT. Treatment was performed during three consecutive years.* Results*. After three years of treatment, a similar reduction in nasal symptoms was observed; according to the visual analog scale, there was a decrease from 3.991 ± 0.804 points to 1.634 ± 0.540 in the ultrarush group and from 3.845 ± 0.265 to 1.501 ± 0.418 in the group desensitized using the conventional method (*P* > 0.05). There was also a comparable reduction in the use of relief drugs during pollen season and an increase in the serum concentration of IgG4 to tree pollens. No significant differences in the safety profile were observed.* Conclusion*. An administration schedule of ultrarush SIT with Purethal Trees is a safe treatment in preliminary observations. This therapy is comparable with conventional administration of SIT in the field of efficacy and safety.

## 1. Background

Specific immunotherapy (SIT) is the common therapy method used in contemporary allergology. The efficacy and safety of SIT are discussed. Allergen-specific immunotherapy has been found to be effective in reducing symptoms of allergic rhinitis, especially in patients with house dust mite and pollen allergies. However, there is a difference in opinions as to whether SIT significantly improves asthma symptoms and about the long-term effects of SIT [1–5]. A new specimen for immunotherapy and an easier administration method (sublingual immunotherapy) aim to increase the efficacy and widespread use of this type of treatment [2, 5]. The accelerated administration schedule of SIT using cluster therapy or ultrarush is available mainly for venom allergies and sometimes for pollen allergies. However, there is insufficient information about the possibility of using these methods for other allergies [6, 7]. This study evaluates SIT with Purethal Tree (birch, hazel, and alder) chemically modified allergens at a concentration of 20 000 BAU (Bioequivalent Allergy Unit)/1 mL for tree pollen to assess the treatment's efficacy and safety.

## 2. Materials and Methods

Twenty-two patients with tree pollen (birch, alder, and hazel) allergies, which manifest as seasonal rhinoconjunctivitis without bronchial asthma, were included in the study. Their allergy was confirmed using the skin prick test (SPT; HAL Allergy, Holland) and a concentration of pollen-specific IgE (sIgE) against birch, alder, and hazel. A positive SPT result was defined by the presence of a wheal with a diameter greater than 3 mm and with a histamine wheal greater than 5 mm. Tests of serum-specific IgE concentrations were performed using the Pharmacia CAP System FEIA (ThermoFisher, Sweden) immunoenzymatic method. The results of these assays were evaluated according to the manufacturer's instructions [8]. A serum concentration of IgE greater than 0.75 IU/mL was confirmed as a positive result. Patients with concomitant bronchial asthma, allergy to grass pollens, house dust mites, Alternaria, or Cladosporium and those with contraindications to SIT were excluded from the study. Asthma was excluded based on a negative history, an examination, and a negative bronchial reversibility test according to Global Initiative for Asthma (GINA) [9].

All patients were randomized and divided into two groups:

- Group A received a conventional administration schedule of SIT with Purethal Trees as preseasonal therapy (October–January): the first dose of 0.1 mL, a second dose of 0.2 mL, and a third dose of 0.4 mL every week, and doses four, five, six, and seven of 0.5 mL every two weeks;
- Group B received an ultrarush administration schedule of SIT with Purethal Trees as follows: the first dose of 0.1 mL, the second dose of 0.2 mL, the third dose of 0.4 mL, and doses four, five, six, and seven of 0.5 mL every 15 minutes (December or January). A comparison of the groups is shown in Table 1.

All patients received immunotherapy for three consecutive years (2010–2012).

Group A included 10 patients (four women and six men) with a mean age of 20.9 ± 4.2 years, and Group B included 12 patients (six women and six men) with a mean age of 21.9 ± 3.2 years. Groups were homogenous in the number of patients and age. All patients in Group A and 11 patients (92%) in Group B completed courses of SIT. One subject did not complete the course of SIT due to lack of cooperation.

### 2.1. Monitoring of Symptoms

All patients described nasal (nasal discharge, blockage, sneezing, and itching), throat, bronchial, and eye allergy symptoms during the 2009 pollen season, a year before starting SIT, and in 2010, 2011, and 2012 during SIT. The patients' allergic rhinitis symptoms were monitored using a 7-point visual analog scale (VAS, published by the Joint Task Force and modified by ARIA) for grading the severity of nasal and nonnasal symptoms in addition to mental function and quality of life changes [10]. The scale was based on the patients' diaries completing during the tree pollen season. All score results were averaged for each tree pollen season (February to May).

### 2.2. Monitoring of Symptomatic Therapy

Patients with allergic symptoms used 5 mg of desloratadine per day or 5 mg of levocetirizine per day, nasal glucocorticoids (fluticasone propionate), and eyes drops (ebastine) as needed. If other drugs were not effective, patients used methylprednisolone (4 mg per day). As symptoms appeared, all patients first used nasal and/or eye drops and then utilized other drugs as necessary. Patients recorded a diary score to monitor daily drug use (one point: nasal or eye drops used at least once per day, two points: therapy as above and one tab of an antihistamine at least one time per day, and three points: therapy as above and one tab of methylprednisolone at least once per day).

### 2.3. Serum IgG4 Measurements

Serum concentrations of IgG4 against birch, alder, and hazel pollens in the blood serum were determined before and after three years of SIT using a four-layer sandwich ELISA test in accordance with procedures used by Lai et al. [11]. Anti-IgG4 monoclonal mouse (Alk Abello A/S) and peroxidase-labeled anti-mouse IgG antibodies were used. The IgG4 concentration was read at 380 nm (CEC 340 Kinetics reader BioTek Instruments Inc., Winooski, VT, USA).

### 2.4. Statistical Analysis

Statistical analysis was performed using Statistica version 8.2 (Soft Pol, Poland). Student's *t*-test was used for unrelated pairs to estimate the significance between drug use and symptoms. The Wilcoxon test was used for all other analyses, and a level of *P* < 0.05 was considered to be statistically significant. All subjects signed agreements to participate in the study. This trial was approved by the Bioethical Committee at the Medical University of Silesia in Katowice, Poland.

## 3. Results

Group characteristics are shown in Table 2.

After three years of SIT, comparable effects were observed in both groups. A statistically significant reduction (*P* < 0.05) in nasal symptom scores during tree pollen season was found in Group A (3.991 ± 0.804 to 1.634 ± 0.540 points) and Group B (3.845 ± 0.265 to 1.501 ± 0.418). Nasal score values during all tree pollen seasons are shown in Table 3.

A statistically significant reduction was found in the scores of other allergic symptoms (*P* < 0.05). Scores in Groups A and B fell from 4.127 ± 0.652 to 2.752 ± 0.136 and from 3.845 ± 0.265 to 2.331 ± 0.109, respectively.

After three years of SIT, use of relief drugs was significantly lower in both groups, as shown in Table 4.

### 3.1. Concentration of Birch, Hazel, and Alder IgG4 during SIT

Before SIT, the mean value of birch IgG4 was 1577 AU/mL (range: 24–1786 AU/mL). The value was significantly higher after three years of SIT, with values of 3566 AU/mL (range: 45–5609 AU/mL) in Group A and 3678 AU/mL (range: 78–6781 AU/mL) in Group B (*P* < 0.05).

Similar trends were observed in alder IgG4. At the start of the study, the mean IgG4 level was 897 AU/mL (range: 22–1877 AU/mL); the value was significantly higher in both groups after three years of SIT, with levels of 4888 AU/mL (range: 88–7680 AU/mL) in Group A and of 3998 AU/mL (range: 102–5667 AU/mL) in Group B (*P* < 0.05).

The serum concentration of IgG4 to hazel also increased during SIT, although they were significantly higher than for alder and birch. At the beginning of the study, the mean alder IgG4 value was 790 AU/mL (range: 45–3821 AU/mL). After three years, mean IgG4 levels were 11786 AU/mL (range: 89–16780 AU/mL) in Group A and 10402 AU/mL (range: 88–15090 AU/mL) in Group B. No statistically significant differences in serum IgG4 levels for any analyzed pollens were found between Groups A and B after three years of SIT (*P* > 0.05).

### 3.2. Safety Results

No systemic anaphylactic reactions (I, II, III, or IV degree reactions) occurred in either group during the course of SIT therapy [12]. Of all 211 Purethal Trees injections performed on patients in Group A, erythema or wheals smaller than 5 cm were observed after 23 injections (10.9%) and erythema or wheals greater than 5 cm after seven injections (3.3%). Similar results were observed in Group B: erythema or wheals smaller than 5 cm were observed after 18 injections (7.8%) and erythema or wheals greater than 5 cm after eight injections (3.5%) out of 231 total injections. There were no statistically significant differences between the groups.

## 4. Discussion

SIT has become a widespread and continuously improving treatment. The ultrarush administration schedule method has been safe and effective in treating allergy to hymenoptera venom [2, 6]. Although many studies emphasize the safety and efficacy of SIT on wasp or bee allergies, SIT administration of vaccines has not been widely used in other studies of allergy desensitization [13–19].

Purethal Tree, which was used in this study, has been confirmed as an effective and safe means to treat a cluster [14]. This type of treatment is an indirect form of an administration schedule between conventional SIT and the ultrarush method. Our work confirmed that Purethal therapy using an ultrarush administration schedule before pollen season is similarly safe.

The results of this study quell doubts about the safety of this treatment. A statistically significant reduction in allergy symptoms during pollen season was correlated with the use of the conventional method. However, it was a preliminary study based on a small group of patients.

The study also showed a statistically significant reduction of symptomatic drug use. In the in vitro control treatment, we used IgG4 measurements. Unfortunately, there is no superior method of assessing the effectiveness of SIT. The observed increase in IgG4 levels after three years of SIT may be further evidence that an ultrarush administration schedule of pollen SIT is effective. The available literature includes studies that primarily attempt to accelerate desensitization to allergens using sublingual immunotherapy, although there are a few case studies of SIT injection. Notably, all of these case studies showed positive efficacy and safety profiles [15–18].

The present study was not double-blinded or placebo-controlled. However, the main aim of this study was to compare two methods of administering a vaccine. An important result from this study is the demonstrated safety associated with the ultrarush treatment compared to the conventional method. No patients had systemic reactions during ultrarush treatment, unlike the group with conventional SIT, which supports currently available data [13, 18–21]. However, as this is a new method of treatment and research is still insufficient, ultrarush SIT should be conducted within 24 hours of hospitalization.

In this study, most patients receiving vaccinations using the ultrarush administration schedule method experienced more minor subsequent allergic reactions. This outcome should be confirmed in a larger group of patients. An important observation regarding this study is that none of the patients treated with an ultrarush administration experienced late reactions. Late reactions cannot be ruled out, and further studies on their likelihood are needed.

## 5. Conclusion

An ultrarush administration schedule of SIT with Purethal Trees is a safe treatment in preliminary observations. This therapy is comparable with conventional administration of SIT in terms of efficacy and safety. However, further investigations on larger groups of patients are required.

## Figures and Tables

**Table 1:** Comparison of two different administration schedules of Purethal Tree SIT.

	Group A	Group B
Dose administration—period of time	0.1 mL, 0.2 mL, 0.3 mL, 0.4 mL—every week 0.5 mL—every two weeks, four times	0.1 mL, 0.2 mL, 0.3 mL, 0.4 mL—every 15 min 0.5 mL—every 15 min, four times

Total BAU dose per year of treatment (10 000 BAU/mL)	100 000 BAU	100 000 BAU

Total BAU dose after entire therapy (3 years)	300 000 BAU	300 000 BAU

BAU: Bioequivalent Allergy Unit.

**Table 2:** Study groups characteristics.

	Group A	Group B	*P*
Age	20.9 ± 4.2	21.9 ± 3.2	NS
Men/women	6/4	6/6	NS
Disease duration	4.7 ± 1.5	4.9 ± 1.3	NS
Mean score of nasal symptoms before SIT	3.991 ± 0.804	3.845 ± 0.265	NS
Mean score of other symptoms before SIT	4.127 ± 0.652	4.011 ± 0.407	NS
Mean drug use score in the season one year before SIT	1.661 ± 0.445	1.788 ± 0.652	NS
Serum concentration of total IgE	402.78 ± 54.8	387.66 ± 74.61	NS
Serum concentration of sIgE to birch	32.72 ± 8.77	35.33 ± 6.87	NS
Serum concentration of sIgE to alder	15.92 ± 3.91	18.73 ± 4.61	NS
Serum concentration of sIgE to hazel	28.55 ± 6.12	30.34 ± 5.88	NS

sIgE: specific IgE; SIT: specific immunotherapy; NS: not statistically significant.

**Table 3:** Nasal symptom scores during tree pollen seasons before and during SIT therapy.

*n* = total daily measurements	Nasal symptom scores as mean scores during tree pollen season			
	II–V 2009	II–V 2010	II–V 2011	II–V 2012
Group A *N* = 1209	3.991 ± 0.804	2.441 ± 0.322	1.760 ± 0.454	1.452 ± 0.207
Group B *N* = 1243	3.845 ± 0.265	2.219 ± 0.422	1.834 ± 0.231	1.588 ± 0.367
*P*	NS	NS	NS	NS

NS: not statistically significant.

**Table 4:** Use of relief drugs during tree pollen season.

*n* = number of days requiring drug use	Mean drug use score during tree pollen season			
	II–V 2009	II–V 2010	II–V 2011	II–V 2012
Group A *N* = 776	1.661 ± 0.445	1.018 ± 0.578	0.880 ± 0.250	0.498 ± 0.213
Group B *N* = 752	1.788 ± 0.652	1.109 ± 0.299	0.745 ± 0.321	0.532 ± 0.244
*P*	NS	NS	NS	NS

NS: not statistically significant; *N*: number of days with score assessment.
